# Antireflection coating of barriers to enhance electron tunnelling: exploring the matter wave analogy of superluminal optical phase velocity

**DOI:** 10.1038/s41598-017-13028-5

**Published:** 2017-10-06

**Authors:** Zijun C. Zhao, David R. McKenzie

**Affiliations:** 1 School of Physics, The University of Sydney, NSW 2006 Sydney, Australia; 2 Centre of Excellence for Quantum Computation and Communication Technology, School of Physics, The University of Sydney, NSW 2006 Sydney, Australia

## Abstract

The tunnelling of electrons through barriers is important in field emission sources and in interconnects within electronic devices. Here we use the analogy between the electromagnetic wave equation and the Schrodinger equation to find potential barriers that, when added before an existing barrier, increase the transmission probability. A single pre-barrier of negative potential behaves as a dielectric “antireflection coating”, as previously reported. However, we obtain an unexpected and much greater enhancement of transmission when the pre-barrier has a positive potential of height smaller than the energy of the incident electron, an unfamiliar optical case, corresponding to media with superluminal phase velocities as in dilute free electron media and anomalous dispersion at X-ray frequencies. We use a finite difference time domain algorithm to evaluate the transmission through a triangular field emission barrier with a pre-barrier that meets the new condition. We show that the transmission is enhanced for an incident wavepacket, producing a larger field emission current than for an uncoated barrier. Examples are given of available materials to enhance transmission in practical applications. The results are significant for showing how to increase electron transmission in field emission and at interconnects between dissimilar materials in all types of electronic devices.

## Introduction

The transport of electrons from one medium to another through a potential barrier is of interest in many device applications. For example, electrons are transported across a barrier from a metal to a vacuum in field emission electron sources as implemented in the scanning tunnelling microscope^1^ and in bright electron sources for scanning electron microscopy^2^. Electrons are also transported across barriers between metals and semiconductors in the source and drain contacts of metal-oxide-semiconductor (MOS) devices and from one semiconductor to another in contacts within MOS and many other types of devices. Interconnect–related dissipation is a key constraint in current and future strategies for power reduction in integrated circuits consisting of densely packed components. Current strategies for reducing interconnect dissipation include low resistance materials such as nanotube conductors^3^. However, the inherent resistance at interfaces between materials created by potential barriers remains a contributing factor. In many cases, the height of the barrier is determined by fundamental material properties such as the work functions of the materials. Therefore, it is important to find ways to increase transmission of matter waves through a given barrier of predetermined height and width by supplementing the given barrier with additional potential distributions that act as reflection suppression layers or, in the language of optics, by using an “antireflection coating”. The idea of an antireflection coating is compatible with the natural analogy^4^ between matter waves, governed by Schrodinger’s equation and light waves, governed by the electromagnetic wave equation. It is often required in optics to increase light transmission through a given layer by adding layers to the front and or rear of the given layer. In the cases of interest here, adding a “pre-barrier” at the interface is the required option. Strategies for designing appropriate pre-barrier potentials are suggested by the analogy between matter waves and electromagnetic waves, and this has been a productive way of creating designs for increasing the transmission probability through quantum mechanical barriers^5,6^. Methods for minimizing reflections at interfaces in optics are well advanced using matrix calculation methods such as that of Abeles^7,8^, methods that are widely used for designing wavelength-selective optical coatings^9^. The coating of an optical element with layers of selected refractive index and thickness can be used to suppress reflections, to induce transmission and to create band pass filters. The design of structures that show enhanced transmission for matter waves is also possible and the fabrication of resonant structures with the properties of a Fabry-Perot interferometer^10,11^ consisting of two identical barriers separated by a region of constant potential, has already been implemented in the resonant tunnelling or Esaki diode. The resonant cavity idea is not well suited to antireflection of general barriers, because of its inconvenient reliance on the symmetry of two participating barriers. However, we propose that the optical analogy is worth pursuing further to look for new classes of solutions to the matter wave barrier antireflection problem.

The optical antireflection problem may in some cases involve reducing reflection from a layer of complex refractive index where the imaginary part of the refractive index mediates absorption of light. In that case, the antireflection layer or layers may also have refractive indices that are complex valued, as discussed by Li *et al*. for the enhancement of transmission of light through a metallic film^12^. The analogous case where it is desired to increase matter wave transmission through a potential barrier that may be complex to account for the loss or gain of probability has been discussed following the work of Bender and Boettcher in 1998^13^ who studied the quantum mechanics of pseudo-Hermitian Parity-Time (${\mathscr{P}}-{\mathscr{T}}$) conserving solutions of Schrodinger’s equation. There are interesting examples of enhanced transmission of matter waves through barriers of complex potential where absorption enhanced transmission is possible by the addition of layers of complex-valued potentials^14^. In these cases, probability can be absorbed with the net benefit of reduced reflection and greater transmission.

Here we show that there is one class of matter wave barrier problem that has an unfamiliar optical analogy, but nevertheless enables an enhancement of transmission through a given real-valued potential barrier. The case where the barrier is real-valued is of immediate practical interest. We use a single real-valued potential barrier that is adjacent to and lies on the incident side of the given barrier to act as an antireflection coating. Given the widespread application of antireflection coatings to minimize reflection losses at optical interfaces, the ability to suppress electron reflection at material interfaces has significant potential to reduce interconnect dissipation in semiconductor devices and to increase emission currents in field emission devices for the same applied electric field^15^.

A field emission source is a good example of a device limited by interfacial barrier reflection. There are three environmental conditions that influence the fluxes and energy distributions of electrons undergoing field emission from a metal surface. Thermionic emission of electrons applies for small electric fields and high temperatures and is traditionally described by the Richardson equation^16,17^. At low temperatures and high electric fields, the emission of electrons in the process known as “cold” field emission, is usually described by quantum mechanical tunnelling. Semi-classical approximate solutions of the time independent Schrodinger equation such as the WKB approximation^18^ are often used, as embodied in the Fowler-Nordheim equation for field emission^19^. Murphy and Good unified the theories for cold field emission and thermionic emission to give an approximate theory for the regime of intermediate electric fields and moderate temperature^20,21^. A weakness of these approximate approaches is that the transmission probability is assumed to be unity for all electron energies greater than the barrier height. Such an assumption requires a sudden non-physical transition from quantum to classical behaviour for electrons when their energy equals and exceeds the barrier height. For these reasons, we use in this paper only exact methods for solving the Schrodinger equation, methods that are accurate for particle energies of the same order as the barrier height and either higher or lower than the barrier.

The recent availability of an exact analytical solution of the one dimensional Schrodinger equation for an electron moving in a region of constant potential that encounters a triangular barrier has highlighted the deficiencies of the WKB approximation for describing field emission, especially when the electron energy approaches the barrier height^22^. This exact solution for a matter wave incident on a single triangular barrier has assisted the current research.

The matrix methods of thin film optics have an analogous approach in Schrodinger wave mechanics known as the transfer matrix method^11,23^, an exact approach for calculating transmission of an incident plane wave through a region of spatially varying potential. Using this method, optical thin film interference filter designs have been transferred into multilayer electron wave filters as discussed by Gaylord and Brennan^5^, using layers that take the form of a potential well (a barrier of negative potential) relative to the potential describing the incident medium, in keeping with the analogy between potential in the Schrodinger equation and the refractive index of conventional media well known in optics.

In the following sections, we first examine the transmission of electrons through a rectangular barrier using the transfer matrix method for incident plane waves. We then apply a finite difference time domain method for solving the time dependent Schrodinger equation for incident wavepackets and apply it to enhancing the transmission through a triangular field emission barrier.

## The analogy between Schrodinger wave mechanics and electromagnetic wave propagation

An optical analogy^11^ of Schrodinger waves propagating in one dimension from a region of fixed potential energy *V*_0_ (the incident medium) into a region of varying potential energy *V* can be set up. The region with potential energy *V* has an equivalent refractive index (complex) given by:

1 $$n=\frac{\sqrt{m(E-V)}}{\sqrt{{m}_{0}(E-{V}_{0})}}=\eta +i\kappa $$

where *m* is the effective mass of the electron inside the region of potential energy *V*, and *m*_0_ is the mass of the electron in the incident medium. Note that the incident electron kinetic energy *E* − *V*_0_ is assumed positive. The refractive index in equation (1) is the ratio of the phase velocities of the matter wave in the incident region to the medium region, in agreement with the definition of refractive index for light waves entering a refractive medium from the vacuum. The energy-wave vector dispersion relation is different for matter waves and light waves. The dispersion relation in both cases is determined by the medium through which propagation is taking place and is information that is required as input, in addition to the governing equation (electromagnetic wave equation or Schrodinger equation). For light waves, the energy-wave vector dispersion relation is determined by the frequency dependence of the dielectric function of the medium and for matter waves, the effective mass of the particle through the energy-momentum relation determines the dispersion. The dispersion relation for the refractive index with frequency follows from the energy-wave vector dispersion and is in general very different for matter waves than for light waves.

**Figure 1:**
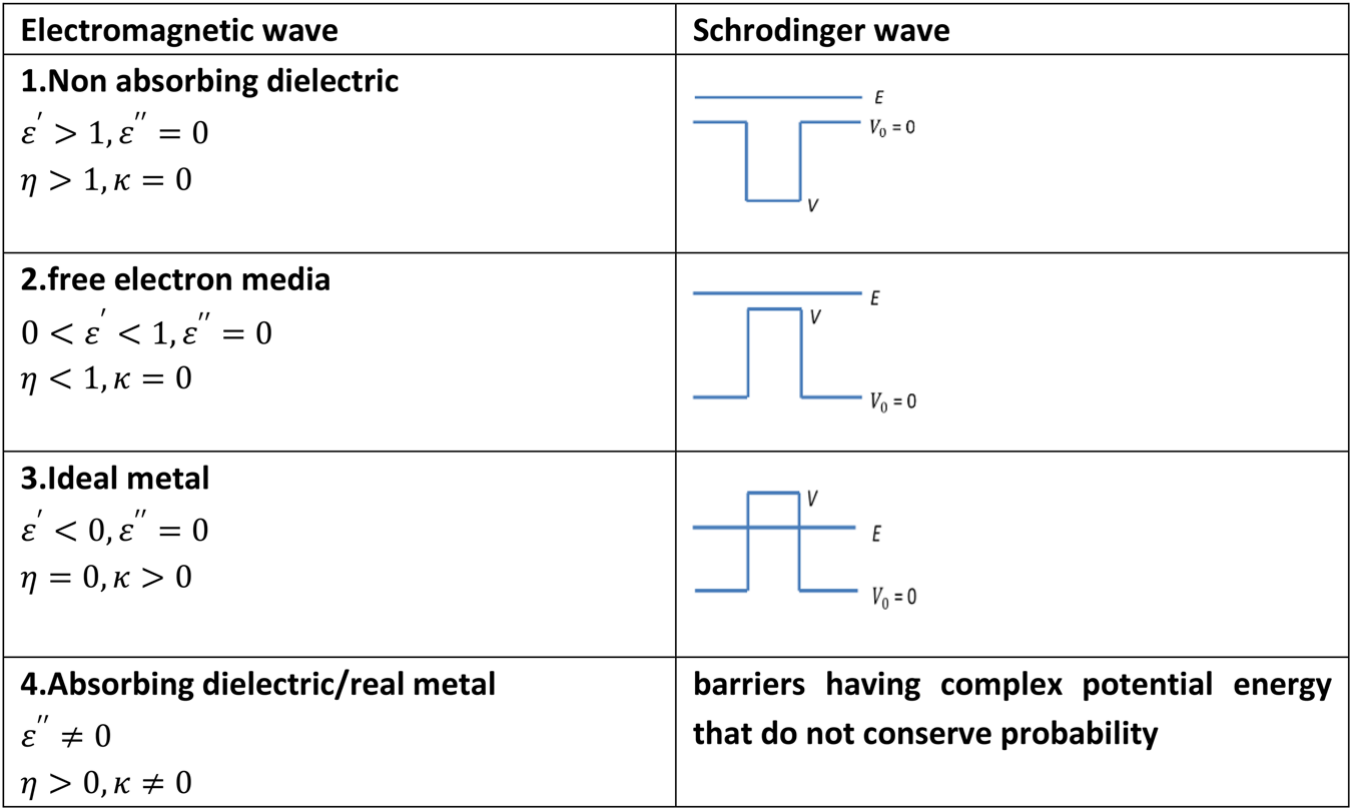
Shows how changing the relative magnitude of total energy *E* and potential energy *V* for a Schrodinger wave (right side) produces analogies with different classes of optical problem (left side). Dielectric permittivity is denoted as ${\boldsymbol{\varepsilon }}={\boldsymbol{\varepsilon }}^{\prime} +i{\boldsymbol{\varepsilon }}^{\prime\prime}$.

The value of the equivalent refractive index of the region of potential energy *V* is either pure real or pure imaginary, depending on whether the sign of *(E-V)* is positive or negative. When *V* < *V*_0_, a potential “well” is formed, *(E-V)* is positive and the layer is analogous to a dielectric layer with a positive real refractive index greater than one. When *V* > *E*, *(E-V)* is negative (i.e. there is no classical transmission) and the layer is analogous to a “perfect metal” with a positive, purely imaginary refractive index. For both of these cases, where the region of potential energy *V* is analogous to a purely real or purely imaginary refractive index, the analogous dielectric permittivity $\varepsilon =\varepsilon ^{\prime} +i\varepsilon ^{\prime\prime} ={n}^{2}=({\eta }^{2}-{\kappa }^{2})+i(2\eta \kappa )$ is real and either positive or negative. Figure 1 shows these cases, as case 1 and case 3 together with their optical analogies. Case 2, where *E* > *V* > *V*_0_ is possible for matter waves but is unfamiliar in optics. The condition 0 < *η* < 1 is usually associated in optics with a strong resonant absorption in an anomalous-dispersive medium where $\kappa \ne 0$. However the condition 0 < *η* < 1 with $\,\kappa \approx 0$ is also approached in special cases of anomalous dispersion. These occur when the frequency of the wave is well above all resonant absorption frequencies or when the medium is a dilute free electron system. The former case occurs in the x ray refractive index of solid media containing only light atoms^24^ and the latter case occurs in the weakly ionized upper atmosphere. Case 2 is, as we shall show, an important case for matter waves, but its rarity in optics may be the reason it has been previously overlooked as a method of achieving barrier antireflection for matter waves. In optics, the condition 0 < *η* < 1 leads to phase velocities of waves exceeding speed of light *c*, although the group velocity is invariably less than *c* because of dispersion. Case 4 is familiar for light waves in absorbing media but not for matter waves as it only applies in cases where probability is not conserved as a consequence of the addition or removal of particle probability from the system. Case 4 will not be further pursued here.

There is a wealth of experience with optical antireflection coatings made up of layers of different refractive index. Selecting just one relevant example from a recent paper^25^, Al Shakhs *et al*. have studied the properties of the simple optical system consisting of a bi-layer immersed in vacuum, where one of the layers has the real part of the dielectric permittivity greater than one (dielectric–like) and the other layer has the real part of the permittivity less than one (metal-like). To achieve the enhancement of the optical transmission of the metal-like layer, these authors have shown that either the condition ${\varepsilon }_{1}^{^{\prime} } < 1 < {\varepsilon }_{2}^{^{\prime} }$ or the condition ${\varepsilon }_{2}^{^{\prime} } < 1 < {\varepsilon }_{1}^{^{\prime} }$ must be satisfied. The optimal antireflection condition is achieved when the thickness *d*_1_ of the first layer is related to the thickness *d*_2_ of the second layer by the relation:

2 $${d}_{2}=\frac{1}{2{n}_{2}k}ta{n}^{-1}[\frac{2{n}_{1}{n}_{2}\,\sin (2{n}_{1}k{d}_{1})({\varepsilon }_{1}^{^{\prime} }-1)}{({\varepsilon }_{1}^{^{\prime} }-{\varepsilon }_{2}^{^{\prime} })(1+{\varepsilon }_{1}^{^{\prime} })+(1-{\varepsilon }_{1}^{^{\prime} })({\varepsilon }_{1}^{^{\prime} }+{\varepsilon }_{2}^{^{\prime} })\cos (2{n}_{1}k{d}_{1})}]$$

where ${\varepsilon }_{1}^{^{\prime} }$ ${\varepsilon }_{2}^{^{\prime} }$ are Re(${{n}_{1}}^{2}$) and Re(${{n}_{2}}^{2}$) respectively, and *k* is the free space wave vector $k=\frac{2\pi }{\lambda }$, where $\lambda$ is the wavelength in vacuo.

The optical analogy of case 1 of Fig. 1 has been used for matter wave problems by Gaylord and Brennan^5^, who designed arrays of potential wells with $V < {V}_{0}$ and showed that an array of potential wells behaves for electron waves in the same way as optical dielectric multilayer stacks behave for light, creating electron energy filters of various types.

We now address the problem of antireflection coating of a potential barrier for which *(E-V)* is negative, that is, when there is no classical transmission. This is the analogy of the optical problem of the antireflection coating of an ideal metal layer using a single non-absorbing dielectric layer. The conditions ${\varepsilon }_{1}^{^{\prime} } < 1 < {\varepsilon }_{2}^{^{\prime} }\,$or ${\varepsilon }_{2}^{^{\prime} } < 1 < {\varepsilon }_{1}^{^{\prime} }$ for achieving antireflection were satisfied by Shakhs *et al*. by choosing one layer from case 1 of Fig. 1 and one from either case 3 or case 4, or two from case 4. We note that the conditions are also satisfied by choosing one layer from case 1 and one from case 2. The latter choice is the key step we make in this work.

## Transmission through adjacent rectangular barriers

**Figure 2:**
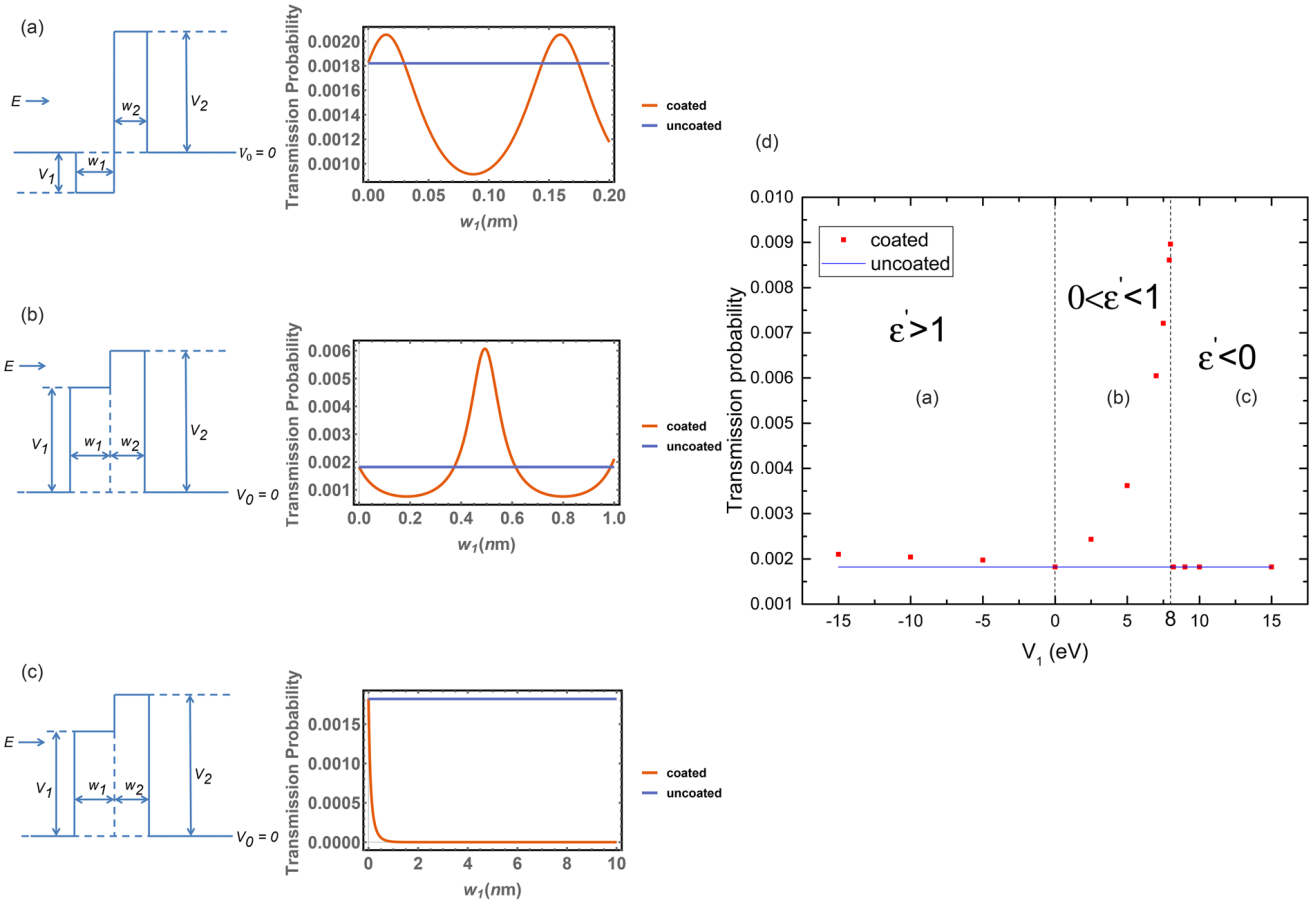
Three approaches to enhancing electron wave transmission through an example barrier of width ${{w}}_{2}=0.5\,{nm}$ and height ${{V}}_{2}=10\,{eV}$ by adding a pre-barrier of width ${{w}}_{1}$ and height ${{V}}_{1}$. The incident electron energy is ${E}\,=\,8\,{eV}$. For (**a**) (**b**) (**c**), the transmission probability is shown on the right as a function of the thickness of the pre-barrier, and the transmission probability for the “uncoated” rectangular barrier is also shown. (a) Using the optical analogy as previously applied, the pre-barrier has negative height ${{V}}_{1}=-10\,{eV}$ and acts as a potential well, giving a marginal transmission enhancement. (**b**)The pre-barrier has positive height ${{V}}_{1}=7\,{eV}$ just below the incident electron kinetic energy and gives a significant enhancement of transmission. This case is unexpected in having an unfamiliar optical analogue. (**c**) The barrier is above the incident electron energy and gives no enhancement of transmission. (**d**) The maximum transmission probability as a function of the pre-barrier height, illustrating each of the three cases (**a–c**). **ε**′ is the real part of the optically analogous permittivity ${\boldsymbol{\varepsilon }}={\boldsymbol{\varepsilon }}^{\prime} +i{\boldsymbol{\varepsilon }}^{\prime\prime}$ for pre-barrier having ${\boldsymbol{\varepsilon }}^{\prime\prime} =0$ for all three cases.

We now calculate the transmission probability for two adjacent rectangular barriers, with the aim of achieving antireflection effects for particle probability. In order to achieve antireflection of given rectangular barrier of height *V*_2_ and width *w*_2_, we apply a rectangular pre-barrier of height *V*_1_ and width *w*_1_. Applying the optical analogy, the height *V*_1_ and width *w*_1_ need to be chosen so that the effective refractive index $n$ satisfies the conditions of Shakhs *et al*. discussed in section 2. Guided by the work of Gaylord and Brennan, one achieves the matter wave analogy to these conditions when ${V}_{1} < 0 < E < {V}_{2}$, where *E* is the incident electron energy, so that the pre-barrier is actually a potential well as shown in Fig. 2(a).

To illustrate this case, we use the transfer matrix method to calculate the transmission probability for a given barrier coupled to a pre-barrier, where the pre-barrier is adjustable. Consider a given barrier height ${V}_{2}=10\,eV$, and width ${w}_{2}=0.5\,nm$. As an instructive first approach we follow the optical analogy of Shakhs *et al*., by choosing a “pre-barrier” (actually a well) of height −*V*_1_ and width *w*_1_. The width *w*_1_ is varied to find the dependence of the transmission for an incident electron with $E=8\,eV$. In this case, the effective mass of the electron is assumed equal to the electron rest mass.

Figure 2(a) shows the dependence of the transmission probability as function the pre-barrier width *w*_1_. The result agrees well with Shakhs *et al*. who found for the analogous optical problem that a positive derivative of transmission probability at zero thickness of the pre-barrier leads to transmission enhancement. However, as we discussed previously, there is another choice of pre-barrier that corresponds to unfamiliar superluminal optical media and therefore has not previously been considered. We show in Fig. 2(b), that this choice leads to a negative derivative of transmission probability at zero thickness of the pre-barrier and also leads to a transmission enhancement. This case of configuration is in Fig. 2(b) where the pre-barrier ${V}_{1}=7eV$. Unexpectedly, the transmission enhancement found is much greater in this case than for the previous case, as shown in Fig. 2(d), which shows that the transmission probability approaches to maximum as *V*_1_ approaches *E*. Therefore a highly favorable condition for transmission enhancement can be specified as $0 < {V}_{1} < E < {V}_{2}$.

The remaining possibility is a pre-barrier where $0 < E < {V}_{1} < {V}_{2}$. We find that no enhancement is possible in this scenario for any width of the pre-barrier as shown in Fig. 2(c) where the pre-barrier is ${V}_{1}=8.2\,eV$.

## Antireflection of a field emission barrier with a single rectangular pre-barrier

In order to calculate the transmission of a general real-valued potential barrier, we use a finite difference time domain (FDTD) numerical algorithm^26^ for solving the time–dependent Schrodinger equation for an incident wavepacket. This approach is suitable for use with barrier potentials of general shape, including the types of barriers encountered in realistic field emission devices that include band bending effects. The use of a wavepacket of finite dimension gives a generality and a flexibility that is not possible in a time independent, plane wave approach. Wave packets enable the description of processes where coherence is important, as for example in solid state devices where excitation arises from short pulses^27^ or from a confined spatial region of a device.

Our FDTD approach has been benchmarked against a recently developed exact solution by Forbes and Deane^22^ for the transmission probability of a plane wave through an idealized representation of a field emission barrier. The exact solution highlights the departures from the WKB predictions and the need to include quantum effects for electrons above the barrier height.

### Transmission through an unmodified triangular field emission barrier

**Figure 3:**
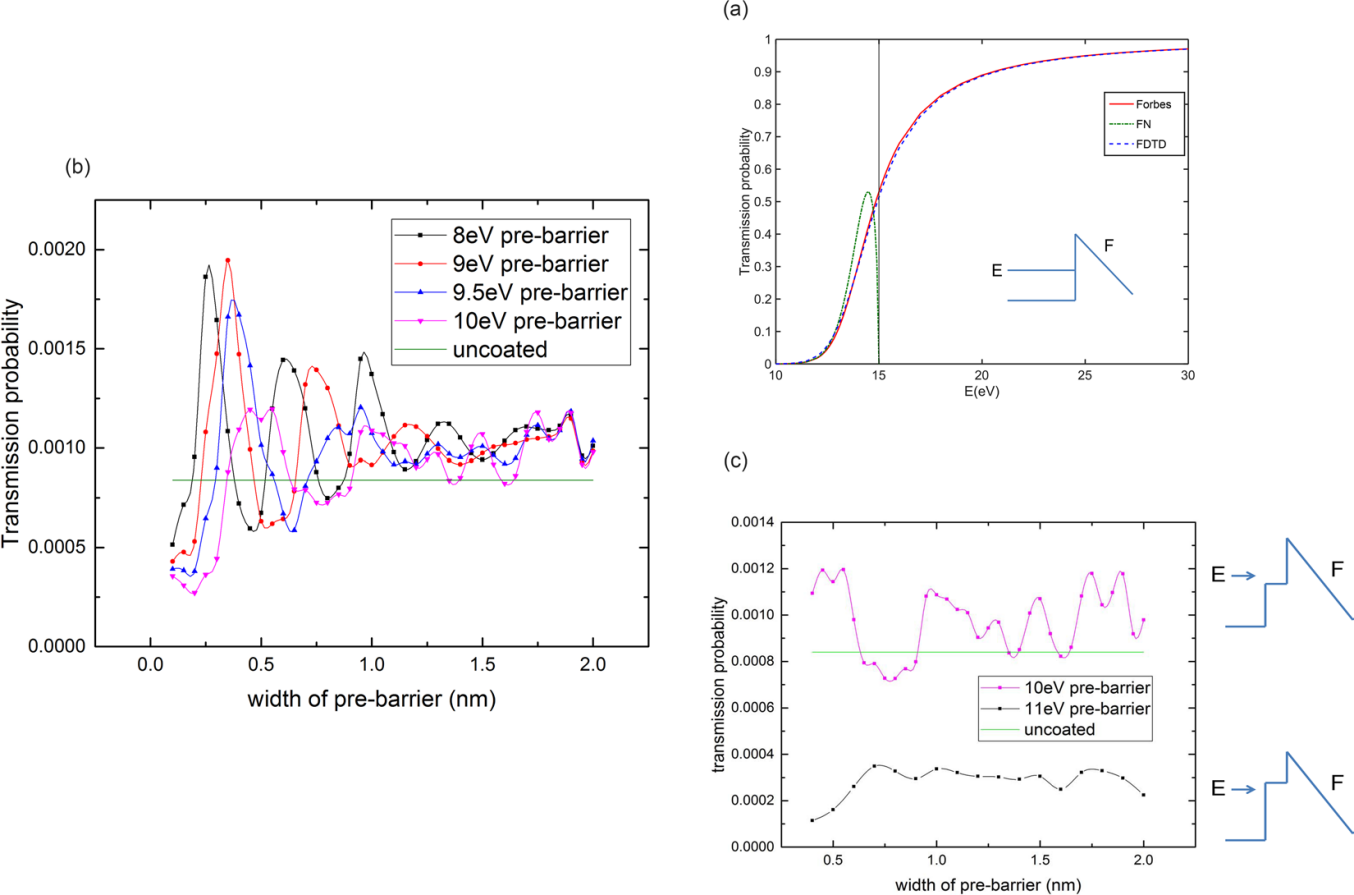
Illustrating the use of a pre-barrier to enhance the transmission of an electron wavepacket through a triangular field emission barrier of height 15 eV. (**a**) Transmission probability is shown as a function of energy *E* of an electron incident on a triangular barrier with a slope determined by the electric field *F* on the vacuum side. The FDTD result compares well with the analytical solution of Forbes and Deane^22^. We also show the analytical solution derived by Fowler and Nordheim (FN) on the basis of the WKB approximation. The vertical line indicates where the electron energy is equal to the barrier height. The FN solution breaks down for electron energies just below the barrier height. (**b**) The transmission probability for four energies below the pre-barrier height as a function of the pre-barrier width, showing the constructive and destructive interference. (**c**) The transmission probability for energies both below and above the pre-barrier height as a function of the pre-barrier width. Enhancement of transmission is only observed in when the incident electron energy is above the barrier height. Here the energy of the incident electron is 10.5 eV.

The triangular barrier is the simplest representation of a field emission barrier, when the barrier edge rounding caused by the image charge effect is not included. The height of the barrier is determined by the work function of the metal, and the slope of the top of the barrier is determined by the electric field in the vacuum outside the metal. The barrier shown in Fig. 3(a) represents a tungsten surface with a work function of 4.5 eV, the zero of potential is the bottom of the energy band, so that the barrier height is 15eV^28^. In the following example, we choose the electric field to be *F* = 8 V/nm.

Figure 3(a) shows the transmission probability as a function of the energy of an incident electron for the triangular barrier shown in the inset. The FDTD method shows good agreement with the analytical solution of Forbes and Deane^22^ for incident plane waves. A wave-packet width of *s* = 2 nm in real-space is used for the FDTD calculation. The result shown in the figure is converged for increasing wavepacket width. Figure 3(a) also shows that the analytical Fowler-Nordheim result based on the WKB approximation overestimates the transmission probability when the electron energy approaches the barrier height, and fails when the energy reaches or exceeds the barrier height.

The result above demonstrates the feasibility of using the FDTD numerical method for finding the transmission probability of triangular barriers for a wide range of incident electron energy both below and above the barrier height, provided that an appropriate choice is made for the wavepacket width. Since there is no available analytic solution for the combination of a rectangular and a triangular barrier we wish to investigate, and because the sloping part of the potential requires inelegant conversion to a stepped potential in the transfer matrix method, we will use FDTD as a tool to calculate the transmission probability in this case.

### Enhancement of transmission of a triangular barrier by a rectangular pre-barrier

In this section, we will use the FDTD method to investigate whether transmission enhancement could be achieved for a given triangular field emission barrier using a rectangular pre-barrier of height *V*_1_ that meets the new condition $0 < {V}_{1} < E < {V}_{2}$, where *V*_2_ is the height of the field emission barrier. The effective mass of the electron is assumed to be equal to its rest mass in this example.

Consider the configuration shown in the inset of Fig. 3(c) where a rectangular pre-barrier of adjustable width and height is placed in front of the triangular barrier. We find when the pre-barrier is higher than the energy of the incident electron, there is no enhancement of the transmission, as was observed in Fig. 2(c) for two rectangular barriers. When the pre-barrier is lower than the energy of the incident electron, there are oscillations in the transmission probability as the pre-barrier width changes. The oscillations indicate interference between the matter waves reflected from the pre-barrier and the triangular barrier.

Figure 3(b) shows by refinement of the pre-barrier height below the energy of the incident electron, we can optimize the maximum in transmission by an appropriate selection of the pre-barrier height and width. In Fig. 3(b) the transmission probability of the uncoated barrier is at least doubled by adding a pre-barrier.

We show in the Supplementary Figure 1 snapshots of FDTD simulations for two cases of incident wavepackets of the same kinetic energy and real space width, one with the optimum transmission of the triangular barrier and one with optimum reflection. In both cases, the interior of the pre-barrier is a site of maximum wave amplitude during the interaction with the incident wavepacket so that the pre-barrier has the effect of confining the probability. The distribution of the confined probability in the forward and reverse directions is dictated by the phase relationships of the waves reflected at each boundary, in much the same way as for an antireflection coating in optics. There is a superficial resemblance to the confinement of probability in Fabry-Perot type resonant tunnelling through two identical barriers separated by a gap, however, the origins of the confinement are different. In our case confinement is caused by the slowing down of the particle over the pre-barrier as its kinetic energy is diminished; in the latter case, there is a standing wave confined by highly reflecting boundaries.

## Achieving practical implementations of electron antireflection barriers

**Figure 4:**
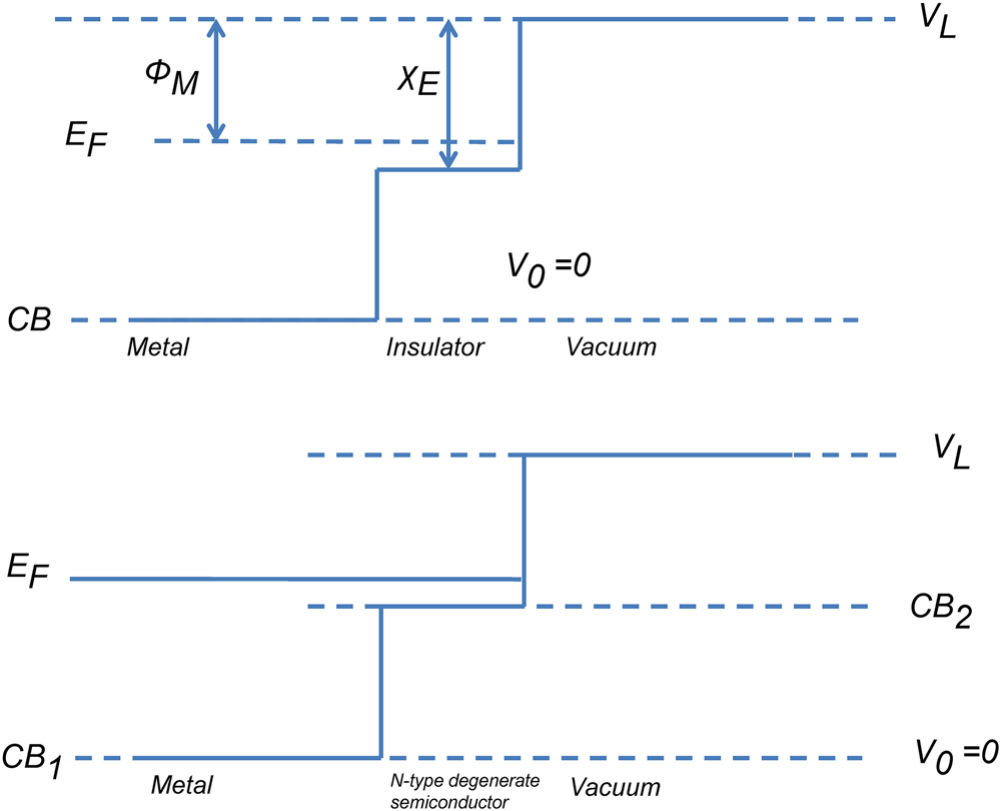
Schematic diagram of band structures of two possible configurations for achieving practical antireflection coatings of a field emission barrier (shown without applied field). The upper diagram represents a metal/insulator/vacuum structure, and the lower one represents a metal/semiconductor/vacuum structure. ***E***_***F***_ represents the Fermi level and *CB* represents the bottom of a conduction band. Potentials are referred to the bottom of the lowest conduction band.

Our strategy for enhancing field emission from a given material is to coat the material with a thin layer of another material. Enhancement of the transmission for electrons at the Fermi energy of the base material will in general yield the best results, since these electrons have the highest transmission for the uncoated barrier. In practice, a barrier of height just below the energy of an electron at the Fermi energy of the incident medium could be achieved by selection of a material with appropriate work function and electron affinity^29–32^. Two possible configurations are shown in Fig. 4. The first case is realized with a metal-insulator-vacuum structure, where the work function ${{\rm{\Phi }}}_{M}$ of the metal is smaller than the electron affinity ${\chi }_{E}$ of the insulator. For example, if the metal is hafnium with ${{\rm{\Phi }}}_{M}=3.9\,eV$, an insulator layer such as ${{\rm{Nb}}}_{2}{{\rm{O}}}_{5}$ with ${\chi }_{E}=4.23\,eV$ and ZnO with ${\chi }_{E}=4.5\,eV$ can both satisfy the criteria. The second possible case could be achieved with metal/degenerate n-type semiconductor/vacuum structure. For a heavily (degenerately) doped n-type semiconductor, the Fermi level is raised so that it lies above the conduction band, and the semiconductor behaves likes a metal, so that when the metal and the semiconductor are in contact, their Fermi levels line up and form the desired band alignments. Doping can be controlled to match the two work functions to avoid excessive band bending effects. For tungsten, an n-type silicon could be used for the antireflection coating.

## Conclusion

In this paper, we have outlined the analogies between the propagation of matter waves in a region of given potential and the propagation of electromagnetic waves in a medium of given refractive index and identify cases that are well known and a case that is previously unexplored. Following a known case of an optical analogy, we have found some transmission enhancement of a given rectangular barrier by the addition of a pre-barrier in the form of a well, in agreement with the findings of Shakhs *et al*. for the optical case of a dielectric antireflection coating of a metal. Pursuing the case when the pre-barrier is a barrier of slightly smaller height *V*_1_ than the electron energy, specified as $0 < {V}_{1} < E < {V}_{2}$, a highly favorable condition for transmission enhancement of a given rectangular barrier of height *V*_2_ was found. The optical analogy for this case requires special and unfamiliar superluminal media with refractive index less than unity where either the frequency is well above all bound resonances, or otherwise the medium contains only free electrons. Applying our findings to a triangular field emission barrier, we used the FDTD method for solving the time dependent Schrodinger equation to show an example of a useful enhancement for a factor of at least two that occurs when a pre-barrier of slightly less than the incident electron energy is added as a pre-barrier to the field emission barrier. We show that the pre-barrier confines probability by slowing down the particle, giving a superficial resemblance to the confinement of probability in resonant tunneling barrier structures. We have given some examples of how to realize configurations where a pre-barrier could be useful in realizing enhanced transmission in practice. This work has further significance in that it shows how matter wave ‘optics’ is free from the constraint applying to normal optical media where equivalent refractive indices are greater than unity. The relaxation of this constraint leads to interesting possibilities for the design of barrier systems with optimized transmission and reflection properties.

## Method

### Finite difference time domain numerical method for a general potential barrier

The time-dependent Schrodinger equation is written in the 1D form:

3 $$i\hslash \frac{\partial \psi (x,t)}{\partial t}=-\frac{{\hslash }^{2}}{2m}\frac{{\partial }^{2}\psi (x,t)}{\partial {x}^{2}}+V(x,t)\psi (x,t)$$

where $V(x,t)\,$is the potential energy, and $\psi (x,t)$ is the wave function of the particle of mass *m*.

For convenience, we separate the complex wave function $\psi (x,t)$ into its real and imaginary parts, denoted as ${\psi }_{R}$ and ${\psi }_{I}$. Noting that the complex conjugate of the wave function ${\psi }^{\ast }$ satisfies the following equation:

4 $$-\,i\hslash \frac{\partial {\psi }^{\ast }(x,t)}{\partial t}=-\frac{{\hslash }^{2}}{2m}\frac{{\partial }^{2}{\psi }^{\ast }(x,t)}{\partial {x}^{2}}+V(x,t){\psi }^{\ast }(x,t)$$

we obtain the following coupled equations for the ${\psi }_{R}$ and ${\psi }_{I}$:

5 $$\{\begin{array}{c}\frac{{\rm{\partial }}{\psi }_{R}(x,t)}{{\rm{\partial }}t}=-\frac{\hslash }{2m}\frac{{{\rm{\partial }}}^{2}{\psi }_{I}(x,t)}{{\rm{\partial }}{x}^{2}}+\,\frac{1}{\hslash }\,V(x,t){\psi }_{I}(x,t)\\ \,\\ \frac{{\rm{\partial }}{\psi }_{I}(x,t)}{{\rm{\partial }}t}=\frac{\hslash }{2m}\frac{{{\rm{\partial }}}^{2}{\psi }_{R}(x,t)}{{\rm{\partial }}{x}^{2}}-\frac{1}{\hslash }\,V(x,t){\psi }_{R}(x,t)\end{array}$$

Space and time step number are respectively denoted as ${n}_{x}$ and ${n}_{t}$, with step sizes Δ*x* and Δ*t*, so that:

$$\{\begin{array}{c}\,t=({n}_{t}-1){\rm{\Delta }}t\\ x=({n}_{x}-1){\rm{\Delta }}x\end{array}\,{\rm{w}}{\rm{h}}{\rm{e}}{\rm{r}}{\rm{e}}\,\{\begin{array}{c}{n}_{x}=1,2,3,\ldots ,{N}_{x}\\ {n}_{t}=1,2,3,\ldots ,{N}_{t}\end{array},$$

with *N*_*x*_ and *N*_*t*_ respectively the total number of space steps and time steps. For a given time *t*, we assign:

$${\psi }_{R}(x,t)\to {y}_{R}({n}_{x}),{\psi }_{I}(x,t)\to {y}_{I}({n}_{x})$$

Using the finite difference method in the following form:

6 $$\frac{\partial \psi (x,t)}{\partial t}=\,\frac{\psi (x,t+{\rm{\Delta }}t)-\psi (x,t)}{{\rm{\Delta }}t}$$

7 $$\frac{{\partial }^{2}\psi (x,t)}{\partial {x}^{2}}=\,\frac{\psi (x+{\rm{\Delta }}x,t)-2\psi (x,t)+\psi (x-{\rm{\Delta }}x,t)}{{({\rm{\Delta }}x)}^{2}}$$

We obtain numerical expressions for the updated wave functions in terms of the values at the previous time step.

8 $$\{\begin{array}{c}{y}_{R}{\rm{^{\prime} }}({n}_{x})={y}_{R}({n}_{x})-{C}_{1}({y}_{I}({n}_{x}+1)-2{y}_{I}({n}_{x})+{y}_{I}({n}_{x}-1))+{C}_{2}V({n}_{x}){y}_{I}({n}_{x})\\ {y}_{I}{\rm{^{\prime} }}({n}_{x})={y}_{I}({n}_{x}\,)+{C}_{1}({y}_{R}({n}_{x}+1)-2{y}_{R}({n}_{x})+{y}_{R}({n}_{x}-1))-{C}_{2}V({n}_{x}){y}_{R}({n}_{x})\end{array}$$

The constants ${C}_{1}=\frac{{\rm{\Delta }}t\hslash }{2m{({\rm{\Delta }}x)}^{2}}$ and ${C}_{2}=\frac{e{\rm{\Delta }}t}{\hslash }$ are written in a form to allow potential energy *V* to be expressed in eV.

The FDTD scheme is stable provided the following condition applies^33–35^

9 $${\rm{\Delta }}t\,\ll \,\frac{\hslash }{\frac{2{\hslash }^{2}}{m{\rm{\Delta }}{x}^{2}}+Max(|V|)}$$

The initial condition is set to be a Gaussian wave packet as follows:

10 $$\{\begin{array}{c}{y}_{R}=\exp (-0.5{(\frac{x-{x}_{c}}{s})}^{2})\cos ({k}_{0}(x-{x}_{c}))\\ {y}_{I}=\exp (-0.5{(\frac{x-{x}_{c}}{s})}^{2})\sin ({k}_{0}(x-{{\rm{x}}}_{c}))\end{array}$$

So the initial probability density function is

11 $${{\rm{\psi }}{\rm{\psi }}}^{\ast }={{y}_{R}}^{2}+{{y}_{I}}^{2}=\exp (-\,{(\frac{x-{x}_{c}}{s})}^{2})$$

with normalization $${\int }_{0}^{L}{{\rm{\psi }}{\rm{\psi }}}^{\ast }dx=1$$.

The width of the Gaussian wavepacket is determined by s. The probability of finding the particle within the range of $6\frac{{\rm{s}}}{\sqrt{2}}$ is 99.73%. The spatial boundaries of our numerical simulation must be sufficiently wide to prevent the loss of probability over the simulation time. The criterion we apply is to maintain the centre of the wavepacket at a distance exceeding 3 $\frac{{\rm{s}}}{\sqrt{2}}$ from the simulation boundaries at *x* = 0 and *x* = *L*.

The program was implemented in Matlab. The input parameters that specify the potential are *V(x)* defined over the interval of *x* in (0, *L*). The solution range *L* is determined so that a given wavepacket can be sufficiently evolved while remaining within the range (0, *L*). For example, a convenient choice is $L=1.25(12s+2w)\,\,$for the problem in this paper, where *w* refers to the range of the non-trivial potential. The initial central position of the wavepacket is set so that it does not touch either the boundary of the solution range or the non-trivial potential. We make the spatial step fine enough to ensure that energy conservation is obeyed. Time step size is determined by equation (9) to make sure the FDTD scheme is stable. Specifically, for the tunnelling problem, the wave function is considered to be sufficiently evolved when the final transmission probability has converged, for example, a typical choice used in this paper is to ensure that the change in the probability over the last 50 time steps is less than 10^−6^. In other cases, the evolution time can be adjusted according to the specific question under investigation.

To confirm the reliability of the computational algorithm and the suitability of the choice of free parameters, we use the calculation of transmission through a simple barrier as a test case.

For a particle with energy *E*_0_ incident on a rectangular barrier with width w and height ${U}_{0}$, for ${E}_{0}$ < ${U}_{0}$, the particle has a tunnelling probability:

12 $$T{}_{analytical}=\,\frac{{(2{k}_{1}{k}_{2})}^{2}}{{({{k}_{1}}^{2}+{{k}_{2}}^{2})}^{2}{\sinh }^{2}(w{k}_{2})+{(2{k}_{1}{k}_{2})}^{2}}$$

where $${k}_{1}=\sqrt{\frac{2m{E}_{0}}{{\hslash }^{2}}}$$, $${k}_{2}=\,\sqrt{\frac{2m({U}_{0}-{E}_{0})}{{\hslash }^{2}}}$$.

**Figure 5:**
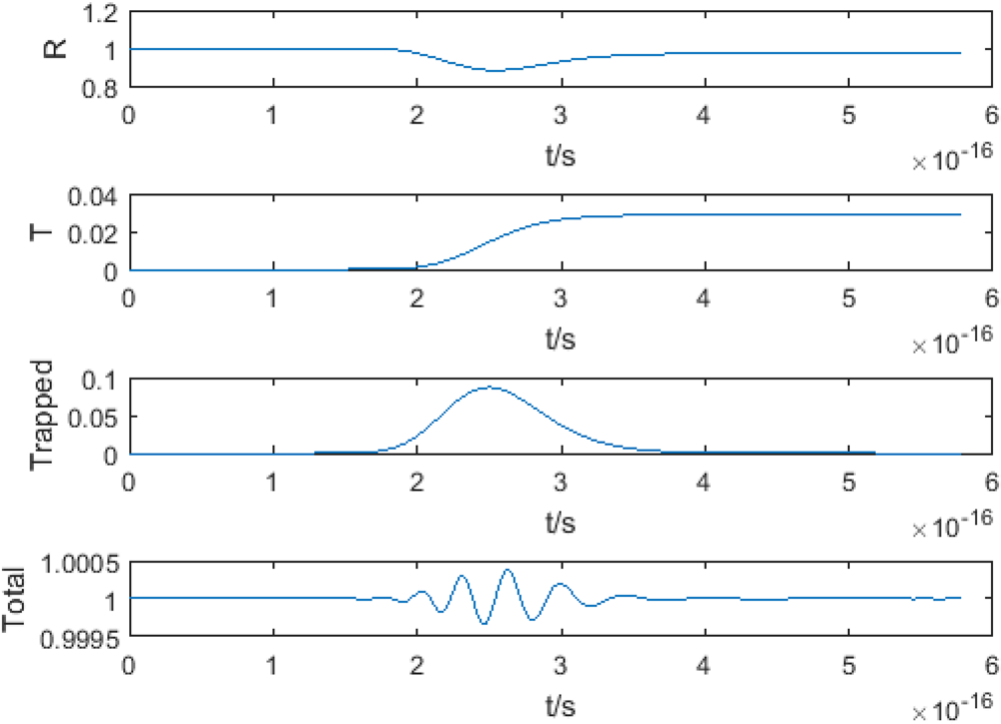
The results of a test of the FDTD method using a rectangular barrier of height 100 eV and width 0.08 nm and an incident Gaussian wavepacket of energy 58 eV and width 0.16 nm. The probability is shown as a function of time for, from top to bottom, the reflected probability *R*, the transmitted probability *T*, the probability trapped in the barrier, *Trapped* and the total probability, *Total*.

This result is readily gained by representing the particle as a sum of plane waves in the regions outside the barrier, and as a sum of attenuated waves inside the barrier. The boundary conditions are that the wavefunction and its first derivative are continuous across the boundaries of the potential. A test case of the FDTD method is shown in Fig. 5.

**Figure 6:**
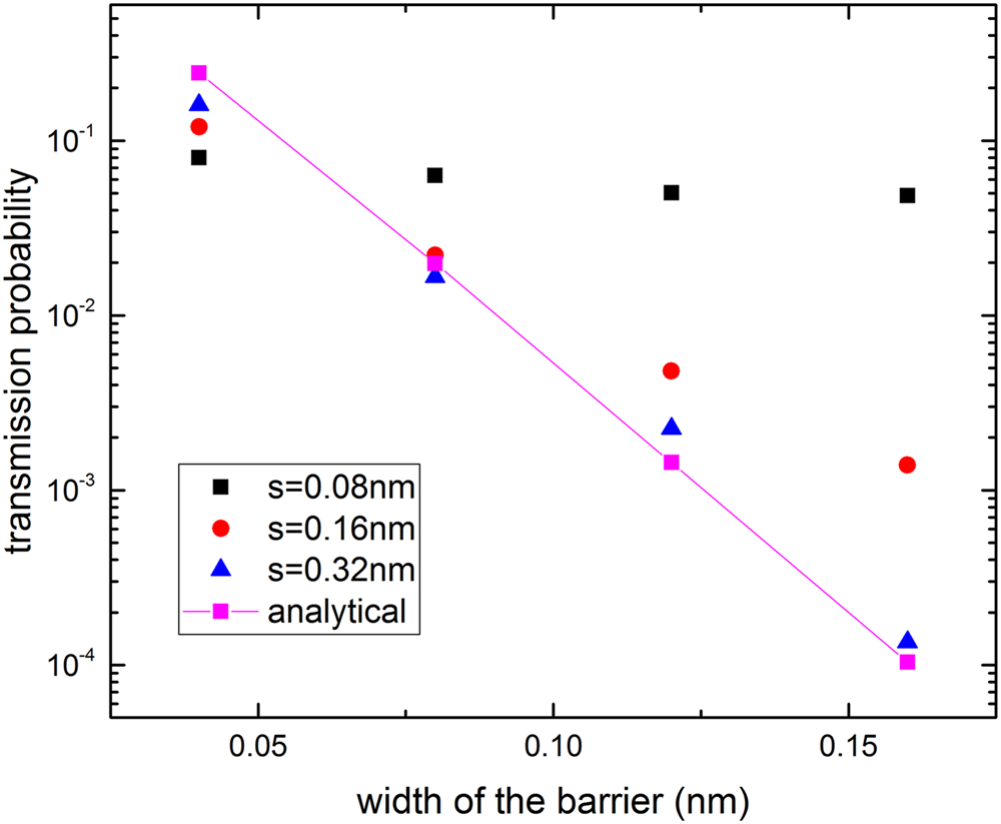
Testing convergence of the transmission probability using the FDTD method to the analytical formula (12) for a plane wave incident on a single rectangular barrier. The transmitted probability of a rectangular barrier (height 100 eV) is shown on a logarithmic scale as a function of the barrier width. The analytical result for a plane wave (solid line) is recovered for wavepacket of width s that is sufficiently large.

As shown in Fig. 6, as the wavepacket gets wider in real space and thus narrower in energy space, the FDTD result approaches more closely to the analytical solution obtained by the plane wave method. The above results of the simple test case show the accuracy of the FDTD method.

### Transfer Matrix Method

By solving the time-independent Schrodinger equation with plane wave approximation, we can write down the wave function for each different potential region separately, and then by matching the boundary conditions for wave function itself and the first derivative, we can find the transfer matrix, and thus find the tunnelling probability.

For *i*th region with potential *V*_*i*_, wave function is written as

13 $${\psi }_{i}={A}_{i}\exp (j{k}_{i}x)+{B}_{i}\exp (-\,j{k}_{i}x)\,({x}_{i} < x < {x}_{i+1})$$

where ${k}_{i}=\frac{\sqrt{2{m}_{i}(E-{V}_{i})}}{\hslash }$, *m*_*i*_ is effective mass, *E* is kinetic energy, *ħ* is reduced Planck constant.

Then we obtain the following form of the generalized 2 by 2 matrix *M*_*i*_ where

14 $$(\begin{array}{c}{A}_{i}\\ {B}_{i}\end{array})={M}_{i}(\begin{array}{c}{A}_{i+1}\\ {B}_{i+1}\end{array})$$

15 $$\{\begin{array}{c}{M}_{i}(1,1)=(\frac{1}{2}+\frac{{k}_{i+1}\,{m}_{i}}{2{k}_{i}\,{m}_{i+1}})\exp (j({k}_{i+1}-{k}_{i}){x}_{i+1})\\ {M}_{i}(1,2)=(\frac{1}{2}-\frac{{k}_{i+1}\,{m}_{i}}{2{k}_{i}\,{m}_{i+1}})\exp (-j({k}_{i+1}+{k}_{i}){x}_{i+1})\\ {M}_{i}(2,1)=(\frac{1}{2}-\frac{{k}_{i+1}\,{m}_{i}}{2{k}_{i}\,{m}_{i+1}})\exp (j({k}_{i+1}+{k}_{i}){x}_{i+1})\\ {M}_{i}(2,2)=(\frac{1}{2}+\frac{{k}_{i+1}\,{m}_{i}}{2{k}_{i}\,{m}_{i+1}})\exp (-j({k}_{i+1}-{k}_{i}){x}_{i+1})\end{array}$$

The total transfer matrix is obtained as matrix products of each individual matrix ${M}_{i}$ as

16 $$[\begin{array}{cc}T(1,1) & T(1,2)\\ T(2,1) & T(2,2)\end{array}]={\prod }^{}{M}_{i}$$

Transmission probability is defined as $T=\frac{1}{|T(1,1){|}^{2}}$ providing that the first and the last region have the same effective mass *m*.

The analytical results based on the transfer matrix method for the transmission probability of the two adjacent rectangular barriers were derived and evaluated using *Mathematica*.

### Code availability

Computer implementation in Matlab and Mathematica can be obtained from the authors if requested.

## Electronic supplementary material

 Supplementary Information
