# Supplementary material for: Antireflection coating of barriers to enhance electron tunnelling: exploring the matter wave analogy of superluminal optical phase velocity
Source: Sci Rep. 2017 Oct 6;7:12772. doi: 10.1038/s41598-017-13028-5 (PMC5630642; doi:10.1038/s41598-017-13028-5)
Supplement: Supplementary file 1 — Supplementary Information [file 41598_2017_13028_MOESM1_ESM.pdf]

Antireflection coating of barriers to enhance electron tunnelling: exploring the matter wave analogy of superluminal optical phase velocity

Zijun C Zhao and David R McKenzie*

School of Physics, The University of Sydney, NSW 2006, Sydney, Australia

Centre of Excellence for Quantum Computation and Communication Technology, School of Physics, The University of Sydney, NSW 2006, Sydney, Australia

Email: david.mckenzie@sydney.edu.au

The figures below have been produced using the FDTD method and show how the pre-barrier confines probability by slowing down the particle. The barrier subtracts kinetic energy, leading to large amplitudes in the real and imaginary parts of the wavefunction in the pre-barrier region of the more slowly moving particle. The confinement of probability happens whether or not the total barrier transmission is enhanced or suppressed. The distribution of the probability between the forward and reverse directions is determined by the phase relationships of the transmitted and reflected waves at the interfaces between boundaries. Enhanced transmission happens when the interference is favourable to the forward propagating wave, in much the same way as happens in an optical interference coating used for suppressing reflection from an interface.

(a)

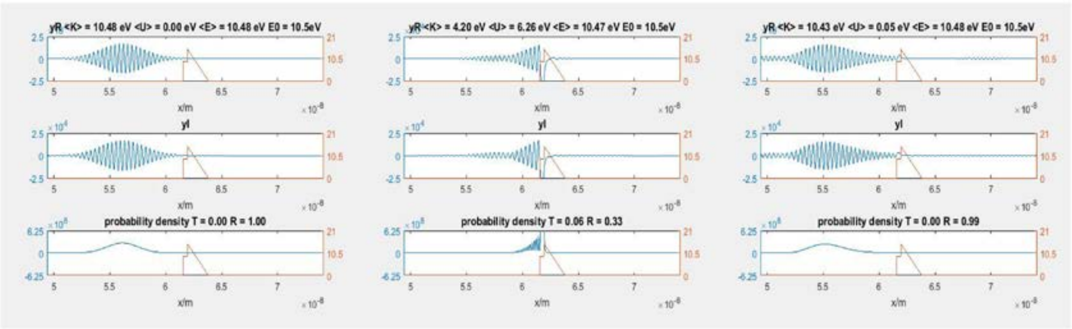

(b)

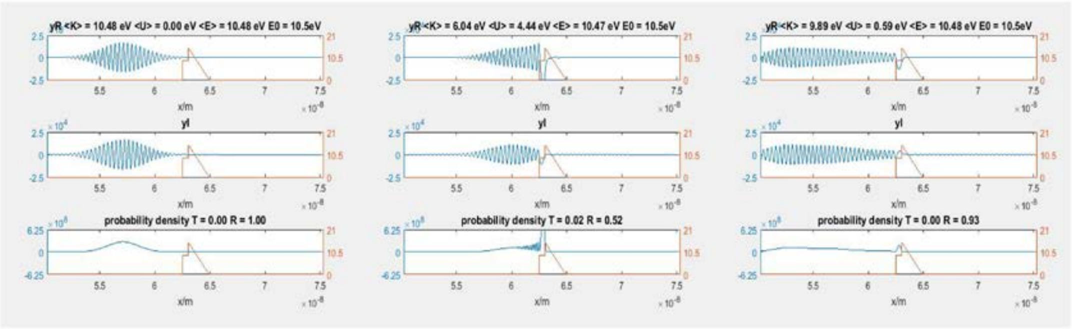

Figure B1 Snapshots of FDTD simulations for wavepackets interacting with a triangular field emission barrier fitted with a pre-barrier of height below the incident electron kinetic energy. The first set of figures refers to a pre-barrier of height 9eV and thickness 0.35nm that enhances transmission. The second set of Figures refers to a pre-barrier of the same height but with thickness 0.55nm that suppresses transmission. For each sub figure, yR represents the real part of the wavepacket shown in the top panels, Real time kinetic energy is denoted as <K>, real time potential energy is denoted as <U>, real time total energy is denoted as <E>, initial kinetic energy of the electron is denoted as E0; yI represents the imaginary part of the wavepacket in the middle panels; probability density is shown in the bottom panels and transmitted probability and reflected probability are shown as *T* and *R* respectively.
